# Offensive Sequences in Youth Soccer: Effects of Experience and Small-Sided Games

**DOI:** 10.2478/hukin-2013-0010

**Published:** 2013-03-28

**Authors:** Carlos Humberto Almeida, António Paulo Ferreira, Anna Volossovitch

**Affiliations:** 1 SpertLab, Faculty of Human Kinetics, Technical University of Lisbon, Estrada da Costa, 1495-688 Cruz Quebrada, Lisbon, Portugal.

**Keywords:** association football, constraints-led approach, performance, skill acquisition

## Abstract

The present study aimed to analyze the interaction and main effects of deliberate practice experience and small-sided game format (3 vs. 3 and 6 vs. 6 plus goalkeepers) on the offensive performance of young soccer players. Twenty-eight U-15 male players were divided into 2 groups according to their deliberate practice experience in soccer (i.e., years of experience in federation soccer): Non-Experienced (age: 12.84 ± 0.63 years) and Experienced (age: 12.91 ± 0.59 years; experience: 3.93 ± 1.00 years). The experimental protocol consisted of 3 independent sessions separated by one-week intervals. In each session both groups performed each small-sided game during 10 minutes interspersed with 5 minutes of passive recovery. To characterize the recorded offensive sequences we used the Offensive Sequences Characterization System, which includes performance indicators previous applied in other studies. No interaction effects on the offensive performance were found between both factors. Non-parametric MANOVA revealed that the factor “experience level” had a significant effect (p<0.05) on performance indicators that characterize the development of offensive sequences, especially in 6 vs. 6 + GKs. While experienced players produced longer offensive sequences with greater ball circulation between them, the non-experienced participants performed faster offensive sequences with a predominance of individual actions. Furthermore, significant differences were observed (p<0.05) in the development and finalization of offensive sequences within each group, when comparing small-sided game formats. Evidence supports that small-sided games can serve several purposes as specific means of training. However, the manipulation of game format should always consider the players’ individual constraints.

## Introduction

In the scope of constraint-led approach, sport performance and skill acquisition emerge from the interaction of constraints pertained to the players, the task and the environment (Handford et al., 1997; Davids et al., 2005; Williams and Hodges, 2005). There have been several attempts to explain how the manipulation of practice constraints influences physical conditioning and/or sport performance (Hill-Haas et al., 2011; Dellal et al., 2011a), however, none of these studies tried to analyze the interaction effects between individual and task constraints on the development of expertise in soccer.

The individual constraints refer to intrinsic characteristics of a person, such as body morphology, chronological and biological age, fitness, skill or experience levels, perceptual and cognitive development, and others (Williams and Hodges, 2005). These unique features play a relevant role in the way a player interacts with external constraints in a specific performance context (Davids et al., 2006). Some studies sustain that capacities and knowledge of skilled players are essentially related to the time spent on training and quality of sport-specific practice, rather than to the maturation process (Ward and Williams, 2003; Vaeyens et al., 2007; Ford and Williams, 2012). One of these works was developed by Helsen et al. (1998), who examined the historical practice profiles of professional, semi-professional and amateur soccer players. These authors concluded that the amount of time spent on collective training was the factor that most distinguished the three skill levels. In a recent review, Ericsson (2006) suggested that the effects of mere experience differ greatly from those of deliberate practice (i.e., activities specifically designed to improve performance). We can assume that the time spent on these types of activities, which can be defined as deliberate practice experience, can be an important component of youth soccer developmental programs. As children gain experience with age and exposure to the sport, they probably develop and refine specific structures of knowledge and motor skills that make them technically and tactically more competent in their game performance.

On the other hand, at present, the researchers take more of an interest in the use of small-sided games (SSGs) in training. Previous studies have pointed out that SSGs are an efficient strategy to increase players’ specific practice time, and to improve physical capacities and technical skills within a major tactical involvement (Reilly, 2005; Duarte et al., 2009). Regardless of their age or skill/experience level, participants of the various studies reviewed performed a greater number of ball possessions, ball touches, dribbles, passes, shots, goals, tackles, interceptions, ball recoveries, and “off-the-ball” movements, when exposed to smaller game situations (Capranica et al., 2001; Jones and Drust, 2007; Hill-Haas et al., 2009; Duarte et al., 2009; Katis and Kellis, 2009; Kelly and Drust, 2009; Casamichana and Castellano, 2010). Hence, the game format presented to players constrains them to solve specific game problems with implications on the individual and collective actions that are performed (Davids et al., 2005; Dellal et al., 2011b; Almeida et al., 2012). Nevertheless, it is not clear how players with different levels of experience respond to similar practice tasks. Are there important differences in their offensive performance?

Given the world-wide popularity of soccer compared to other team sports, there is a surprising lack of scientific interest in the relationship between the deliberate practice experience and the manipulation of task constraints in the development of game actions (for an exception see Ford and Williams, 2012). Therefore, to better understand the attacking playing patterns emerging during SSGs performance, this study aimed to analyze the interaction and main effects of both deliberate practice experience and SSG format on performance indicators that characterize the offensive sequences produced by teams of young soccer players.

## Material and Methods

### Participants

Twenty-eight U-15 males were selected as participants. Ethics approval for this study was granted by the researchers’ faculty scientific board and parental written consent was received prior to all experimental procedures. Participants were divided into 2 groups according to their deliberate practice experience in soccer; a questionnaire was applied to determine the years of experience in soccer. The Non-Experienced group (N-Exp) consisted of 14 participants (age: 12.84 ± 0.63 years) selected from physical education classes with no deliberate practice experience in soccer. The Experienced group (Exp) was formed by 14 participants (age: 12.91 ± 0.59 years) selected from a U-15 club team competing at the Portuguese regional level and with 3.93 ± 1.00 years of deliberate practice experience in soccer. Each group was divided into 2 balanced teams in the 6 vs. 6 + GKs games: 1 goalkeeper, 2 defenders, 3 midfielders and 1 forward. In the 3 vs. 3 + GKs games, the same goalkeeper, one of the defenders, one of the midfielders and the forward took part in the experiment; the defender and the midfielder were randomly selected. Matches were always contested by teams with the same experience level.

### Procedures

Both groups completed 3 independent sessions separated by one-week intervals. All matches were played in the afternoon, during the same hours of the day (between 3:00 and 5:00 p.m.), and under mild temperatures. In each session both groups performed the 2 SSGs – 3 vs. 3 + GKs (4-a-side) and 6 vs. 6 + GKs (7-a-side) – during periods of 10 minutes interspersed with 5 minutes of passive recovery. Matches were divided into two 5-min halves with 1-min intervals for midfield exchange. Game duration was based on coach experience, taking into account that each 5-min half does not correspond to the effective playing time due to the stoppages (e.g., fouls, goals, throw-ins, goal kicks, etc.) that normally occur in soccer matches (Casamichana and Castellano, 2010). Time procedures employed in previous research concerning SSGs in soccer and futsal were also considered (Jones and Drust, 2007; Duarte et al., 2009; Casamichana and Castellano, 2010; Almeida et al., 2012).

The total number of SSGs performed was 12 (i.e., 3 of 3 vs. 3 + GKs and 3 of 6 vs. 6 + GKs games per group), and they were presented in a random order in each session. Whereas the 3 vs. 3 + GKs game is frequently employed in training in youth soccer, the 6 vs. 6 + GKs game is an official competitive game format used in younger age categories across several countries. In the present study, these SSGs were played in pitches with 46 × 31 m and 62 × 40.4 m (length × width), respectively. The relative space available for each player (ratio of pitch area per player), and the dimensions of goalkeeper areas (9 × 24 m; length × width) were similar in both game situations. Prior to game practice, participants performed a 10-min standardized warm-up. Game situations were explained before the first session and participants were asked to play at their best level in order to succeed in SSGs. All the official rules of soccer were implemented apart from the offside rule.

The research took place in a sports park equipped with artificial turf, having all the necessary conditions for soccer practice. Two 7-aside goals were used with the official dimensions of 6 × 2 m, 12 5-size balls properly prepared for the matches, as well as game shirts to differentiate teams. The match time was clocked continuously with a watch (Nike WR0066-001 Triax, Nike^®^ Inc., U.S.A.); one assistant was placed in each touchline of the pitch to reduce the time loss when the ball went out of play. Refereeing was carried out by a neutral assistant. The experimental sessions were only held if the pitch surface was perfectly dry. There was no audience and assistants were not allowed to concede any instructions or feedbacks during practice.

### Data Collection and Analysis

Data were always collected with a digital video camera (Sony DCR-SR77, Sony^®^ Corporation, China) set up on a tripod 42 m from the closest corner of the larger pitch, and at an elevation of 15 m. The camera remained fixed on the tripod, after obtaining a clear and comprehensive plan of the entire pitch surface in each SSG. Data were recorded on a Microsoft Office Excel 2007 sheet (Microsoft^®^ Corporation, U.S.A.) and, finally, exported to the SPSS Statistics, version 17.0 (SPSS^®^ Inc., U.S.A.).

The units of analysis were the offensive sequences, which were understood as the execution of one or more individual and/or collective tactical-technical actions, defined according to criteria of the beginning and end of ball possession. The characterization of each offensive sequence was carried out through a hand notation analysis system specifically designed for this purpose – Offensive Sequences Characterization System (OSCS) – and includes performance indicators previously applied in other investigations. Thus, the offensive sequences were characterized in terms of Duration of ball possession (Hughes and Churchill, 2005; Almeida et al., 2012), number of Players involved (Almeida et al., 2012), number of Ball Touches (Dellal et al., 2011b; Almeida et al., 2012), number of Passes (Hughes and Bartlett, 2002; Hughes and Franks, 2005; Redwood-Brown, 2008), number of Shots (Hughes and Bartlett, 2002; Hughes and Franks, 2005; Almeida et al., 2012), and Result of the Offensive Sequence (Almeida et al., 2012). This last performance indicator is a nominal variable and assumes one of three forms: i) *total success*: goal scored; ii) *partial success*: shot on goal without scoring, the ball hits the goalposts/crossbar or is saved by the goalkeeper or another player near the goal line; iii) *unsuccessful*: shot goes wide of the goal, shot intercepted by an opponent, and loss of ball possession. Since these performance indicators directly derived from the behaviors observed through the system, they are from now on designated as simple indicators.

Moreover, the comparison of performances between teams or team members is often facilitated if the performance indicators are expressed as ratios (Hughes and Bartlett, 2002). Thus, we also analyzed composite indicators, which were obtained by dividing 2 simple indicators: Players involved/Duration (of ball possession), Ball Touches/Duration, Passes/Duration, Ball Touches/Players involved, Passes/Players involved, Passes/Ball Touches, and Goal/Shots. Additionally, the simple and composite indicators were grouped in 1 of the 2 levels configured to characterize the offensive sequences: development and finalization.

### Reliability

Prior to the study, an observation protocol was completed in order to determine the intra-operator reliability when collecting data with the OSCS. The protocol included 2 analysis sessions spaced at least 7 days apart to prevent any learning effects influencing the data (Jones and Drust, 2007). In both sessions (test and retest), data corresponding to 20% of the total images’ sample (80 offensive sequences) were observed and notated. First, the level of agreement for simple performance indicators was determined using the number of exact agreements observed between each of the 2 analysis sessions. The percentage agreement between test and retest measures provides an indication of the consistency of the data (Kelly and Drust, 2009). Results showed high percentages in all performance indicators analyzed (from 85 to 100%). Then, this approach was supplemented by the calculation of the weighted version of kappa statistic to evaluate the reliability of both assessments in all performance indicators (Robinson and O’Donoghue, 2007). There was a very good strength of agreement (O’Donoghue, 2010), since kappa values (κ) ranged from 0.84 to 1.0; these results testify the intra-operator reliability in using the system.

## Statistical Analysis

The effects of the “experience level” and “SSG format” on offensive performance indicators were primarily studied through descriptive statistics (means, standard deviations, and absolute frequencies). Then, after the rejection of the multivariate normality assumption (using Kolmogorov-Smirnov tests for each performance indicator) and the homogeneity of covariance matrices (using Box’s M test), non-parametric MANOVAs were applied to assess the interaction and the main effects of both factors on simple and composite indicators. For each MANOVA, partials eta squared (η_p_^2^) were calculated as measures of effect size. As a third step, if the effects of factors on performance indicators were significant, it would be essential to identify in which the significant differences occurred. Multiple Mann-Whitney tests were applied for that purpose. Finally, chi-square tests were selected for the nominal variable of the Result of the Offensive Sequences. For all statistical procedures alpha (α) was set at 0.05.

## Results

In the 12 matches played, an overall of 398 offensive sequences were identified. Table 1 presents the descriptive statistics (mean ± SD) of performance indicators that characterize the offensive sequences produced by players in each SSG.

First of all, the MANOVA models revealed that the interaction effects of both factors (experience level*SSG format) were not significant on simple and composite performance indicators (p=0.72; η_p_^2^=0.007, and p=0.423; η_p_^2^=0.015, respectively). When considering the main effect of the “experience level” in 3 vs. 3 + GKs games, the non-parametric MANOVAs demonstrated significant effect on simple and composite indicators that characterize the offensive sequences (p<0.001; η_p_^2^=0.106, and p<0.001; η_p_^2^=0.108, respectively). Concerning the development of offensive sequences, Mann-Whitney tests exhibited significant differences between groups (N-Exp vs. Exp) in number of: Players involved (p=0.003), Passes (p<0.001), Passes/Duration of ball possession (p=0.005), Passes/Players involved (p<0.001), and Passes/Ball Touches (p<0.001). No differences between experience levels were observed in performance indicators associated to the finalization of offensive sequences.

The application of non-parametric MANOVAs testified that the deliberate practice experience showed a significant effect on simple and composite performance indicators underlying the characterization of offensive sequences in 6 vs. 6 + GKs games (p=0.012; η_p_^2^=0.081, and p=0.024; η_p_^2^=0.07, respectively). The Mann-Whitney test revealed that the offensive sequences differed significantly between both groups in the performance indicators such as Duration of ball possession (p=0.016), Players involved (p=0.004), Ball Touches (p=0.003), Passes (p<0.001), Passes/Duration (p=0.009), Passes/Players involved (p<0.001), and Passes/Ball Touches (p=0.008). No significant differences were observed at the finalization level in any of the performance indicators analyzed.

The factor “SSG format” evidenced a significant influence on simple and composite indicators of the offensive sequences produced by the “N-Exp” group (p<0.001; η_p_^2^=0.151, and p<0.001; η_p_^2^=0.062, respectively). Significant differences were discriminated between SSGs in the following performance indicators: Players involved (p=0.003), Ball Touches/Duration (p=0.003), Ball Touches/Players involved (p=0.005), and Shots (p=0.02). Regarding the “Exp” group, the non-parametric MANOVA reported a significant effect of the factor “SSG format” only on simple indicators (p<0.001; η_p_^2^=0.194). Therefore, the differences between game formats in the “Exp” group were only significant for the Duration of ball possession (p=0.033), Players involved (p<0.001), and Shots (p=0.011).

Table 2 demonstrates a broader perspective on the offensive sequences’ finalization in both experience levels and in each game format.

The statistical inference showed that the distribution of the offensive sequences’ results was homogeneous between groups for 3 vs. 3 + GKs (p=0.478) and 6 vs. 6 + GKs (p=0.236) games. According to an intra-group perspective, statistical evidence indicated that the distribution of the results for this category was not homogeneous in both SSGs for the N-Exp group (p=0.04), although it was homogeneous for the Exp group (p=0.734).

## Discussion

### Experience effects on the offensive performance in each small-sided game

The purpose of this paper was to analyze the interaction and main effects of deliberate practice experience and SSG format on the offensive performance of young soccer players. Although no significant interaction effects were observed between independent variables, results indicate that the experience level and the SSG format significantly influenced the offensive performance of young athletes.

In this investigation, deliberate practice experience refers to the average value of 3.87 years of soccer training for participants from the Exp group. Despite some differences in the aims and methods, data obtained concur with studies of Ward and Williams (2003), Williams and Hodges (2005), and Ford and Williams (2012) who observed that the experience is an aspect which influences the competitive performance that is directly related to the time of practice and competition in soccer. Nevertheless, it seems that it does not require long periods of deliberate practice in order to verify differences in the collective performance comparatively to youngsters who only play the game for enjoyment and fun (i.e., deliberate play).

In both SSGs, the level of experience in soccer had a significant effect on performance indicators which characterize the development of offensive sequences. The observed effect sizes on simple and composite performance indicators are higher than the recommended minimum effect size representing a practical significant effect (i.e., η_p_^2^=0.04), and ranged from small to moderate (Ferguson, 2009). As an individual constraint, the deliberate practice experience should be considered by researchers or coaches when analyzing game performance or designing talent developmental programs in youth soccer.

Experienced participants performed significantly longer offensive sequences, with a greater number of players involved, who, in turn, executed more touches on the ball and more passing actions. The main difference observed in the composite indicators suggests that the Exp group adopted a “possession play” style, which implies a more intensive and variable ball circulation. The non-experienced participants showed a tendency to build offensive sequences through individual actions, possibly trying to quickly explore the defensive disorganization that is common in lower skill levels. In this group, offensive sequences were, on average, shorter and involved fewer players, which is associated with the execution of a lower number of passes. This style of play is usually observed in novice players, whose tendency is to perform individual actions to solve contextual problems during matches.

Williams (2000) stated that the ability to “read the game” distinguishes skilled from less skilled soccer players. Several studies have shown that expert players are faster and more accurate to recognize and recall patterns of play, demonstrate more effective and efficient visual search strategies and make better decisions in competitive contexts (Vaeyens et al., 2007). Since the soccer game is an eminently tactical reality, these aspects of perceptual-cognitive nature are plausible enough to perceive the differences verified in both groups. Especially in 6 vs. 6 + GKs, experienced participants differed even more from the N-Exp group by producing longer offensive sequences with a greater number of ball touches. Mastery of specific motor skills such as ball control, pass or dribble allows teams to maintain ball possession during longer periods and, therefore, to exploit the weaknesses of the opponent defensive organization in the most appropriate moments. Nevertheless, the “possession play” style does not relate exclusively to the superior technical ability of the more experienced players, but essentially to the ability to establish an effective unity of cooperation among team players (Reilly, 2005). From this perspective, “reading the game” must be a quality to develop since the early stages of training with young soccer players.

The differences observed between groups for the indicators that characterize the finalization of offensive sequences were not statistically significant either in 3 vs. 3 + GKs or 6 vs. 6 + GKs games. These results support the investigation of Reilly et al. (2000), which evidenced no significant differences in shooting test between U-16 elite and sub-elite players. The overview of the development and finalization of offensive sequences allows us to infer that the N-Exp group scored more goals using faster offensive methods, predominantly built through individual actions. Instead, the Exp group made more shots and scored fewer goals, executing more passes and involving more players in the process. Finally, we emphasize that the number of passes made in the offensive sequences led to major differences ascertained between groups of participants. This finding prompts us to agree with Redwood-Brown (2008), who mentioned that the execution of accurate passes contributes not only to create scoring opportunities, but also to restrict the possession of the opposite team, and, ultimately, to achieve success in soccer.

### Effects of small-sided games on the offensive performance at each level of experience

The development of sport expertise should be regarded as being influenced by an interaction of internal and external constraints (Araújo et al., 2010). As far as we are aware, the deliberate practice experience (individual constraint) and the manipulating of pitch size and number of players (task constraint) have not been previously analyzed simultaneously. In this study, the configuration of the SSG format manifested a significant effect on the offensive sequences’ characteristics, except for the composite indicators of the Exp group. Partials eta squared values (effect sizes) ranged from small (composite indicators of N-Exp group) to moderate (simple indicators of both groups) (Ferguson, 2009). Such evidence confirms that manipulating task constraints may induce modifications in specific behaviors of players and, thereby, foster the development and improvement of technical, tactical, and strategic skills in team sports (Williams and Hodges, 2005; Almeida et al., 2012).

In terms of the development of offensive sequences, both SSGs differed significantly in the rhythm of intervention on the ball and the number of consecutive ball touches carried by each player, yielding higher values in 3 vs. 3 + GKs. Data from the N-Exp group confirmed findings from other investigations (Capranica et al., 2001; Jones and Drust, 2007; Duarte et al., 2009, Hill-Haas et al., 2009; Kellis and Katis, 2009; Casamichana and Castellano, 2010), in which each player revealed a more effective relationship with the ball in smaller game formats. The Exp group exhibited greater stability in collective performance profiles between both game formats. The duration of ball possession and the number of players involved in the offensive sequences presented higher values in 6 vs. 6 + GKs, which underscore the ability of the experienced teams to keep the ball in larger pitch sizes and involving more players.

Considering the finalization of offensive sequences, the number of shots was significantly higher in 3 vs. 3 + GKs in both groups, although this did not reflect on the effectiveness of shots. The non-experienced participants were more successful in 3 vs. 3 + GKs games, because they scored more goals, executed more shots on goal and produced less unsuccessful offensive sequences in this game format. In 3 vs. 3 + GKs, the experienced players not only concluded more successful (total and partial) offensive sequences, but also ended more unsuccessful offensive sequences. Decreasing the pitch size and players’ number provided an increase in the number of shots and goals. Such facts are consistent with previous studies (Katis and Kellis, 2009; Casamichana and Castellano, 2010), which revealed that players had more opportunities for shooting and scored more goals in smaller game formats.

### Deliberate practice experience and small-sided games: practical implications

Overall, 3 vs. 3 + GKs games ensure that players will get more directly involved in the match, since they contact more often with the ball, execute accurate passes and shoot on goal more frequently. This evidence seems to be especially important for the N-Exp group. The non-experienced participants demonstrated a poorer adaptation to 6 vs. 6 + GKs, explained by differences recorded in the results of offensive sequences and especially by the greater number of unsuccessful offensive sequences in this game format. Our data suggested that the game format clearly affects the quantity and quality of performed actions and, consequently, the offensive sequences’ characteristics. Katis and Kellis (2009) argued also that SSGs can serve several purposes as specific means of training. According to Reilly (2005), since young players need to develop physical abilities, technical skills and decision making in specific performance contexts, it makes sense to use SSGs depending on the age of the participants. However, youth soccer coaches should be aware that age is not a very precise variable to use in the organization of the training task. Even within the same age group, it is possible to note considerable differences in individual and collective performance of players with well distinct skill and experience levels.

In summary, the present paper has evidenced that the deliberate practice experience (individual constraint) and the SSG format (task constraint) do not depend of each other (interaction effect) to influence the offensive performance of young soccer players. Both factors affected independently the characteristics of the offensive sequences. Findings confirm that SSGs can serve several purposes as specific means of training. However, for effective skill acquisition and performance enhancing in youth soccer, the manipulation of pitch size and number of players should always consider the players’ individual constraints. In this sense, smaller game formats seems to be particularly suitable for novice athletes (e.g., children/youngsters without experience and/or with a lower skill level), since they constrain the development of sport-specific skills based on a major involvement with the ball. On the other hand, in larger SSG formats, the number of actions that each player performs on the ball tends to decrease, increasing the number of “off-the-ball” movements and the need to form an effective unit of cooperation with teammates. Such game formats may be useful to practice the specific movement requirements of competitive situations (Hill-Haas et al., 2009), and should be carefully considered as young players improve game understanding and specific motor skills.

Further studies should continue to verify how the task constraints imply the efficacy of the learning process in soccer, aggregating different contexts and players’ experiences. The functional impact, as far as technical and tactical behavioral changes in consequence of pitch size and number of players’ modifications are concerned, should be clarified.

## Figures and Tables

**Table 1:** Mean values (± SD) of performance indicators that characterize the offensive sequences produced by groups in each SSG format.

	**3 vs. 3 + GKs**		**6 vs. 6 + GKs**	

**Performance Indicators**	N-Exp	Exp	N-Exp	Exp
**DEVELOPMENT**				
Duration of ball possession (s)	10.67 (6.53)	12.39 (7.94)^†^	12.28 (9.04)^*^	15.17 (10.1)^*†^
Players involved (n)	2.28 (0.83)^*†^	2.64 (0.85)^*†^	2.8 (1.26)^*†^	3.4 (1.47)^*†^
Ball Touches (n)	7.31 (4.67)	8.47 (5.77)	7.42 (5.45)^*^	9.64 (6.0)^*^
Passes (n)	1.61 (1.48)^*^	2.58 (2.04)^*^	1.83 (1.68)^*^	3.03 (2.5)^*^
Players involved/Duration (n/s)	0.28 (0.18)	0.27 (0.14)	0.29 (0.15)	0.27 (0.11)
Ball Touches/Duration (n/s)	0.72 (0.25)^†^	0.69 (0.22)	0.64 (0.23)^†^	0.66 (0.2)
Passes/Duration (n/s)	0.16 (0.13)^*^	0.2 (0.11)^*^	0.16 (0.11)^*^	0.19 (0.11)^*^
Ball Touches/Players involved (n/n)	3.22 (2.04)^†^	3.04 (1.48)	2.55 (1.22)^†^	2.83 (1.31)
Passes/Players involved (n/n)	0.59 (0.48)^*^	0.87 (0.56)^*^	0.55 (0.36)^*^	0.77 (0.5)^*^
Passes/Ball Touches (n/n)	0.21 (0.16)^*^	0.29 (0.15)^*^	0.24 (0.15)^*^	0.29 (0.15)^*^
**FINALIZATION**				
Shots (n)	0.39 (0.56)^†^	0.5 (0.64)^†^	0.24 (0.53)^†^	0.28 (0.52)^†^
Goals/Shots (n/n)	0.38 (0.48)	0.17 (0.36)	0.29 (0.44)	0.26 (0.44)
**OFFENSIVE SEQUENCES**	**103**	**102**	**107**	**86**

* Significant difference (p<0.05) between experience levels in each SSG format.

† Significant difference (p<0.05) between SSG formats in each experience level.

**Table 2:** Results of the offensive sequences (absolute frequencies) performed by groups in each SSG format.

		**Results of the Offensive Sequences**			**Total**
		Total Success	Partial Success	Unsuccessful	
**N-Exp**	3 vs. 3 + GKs	14	7	82	**103**
	6 vs. 6 + GKs	7	2	98	**107**
**Exp**	3 vs. 3 + GKs	9	10	83	**102**
	6 vs. 6 + GKs	6	6	74	**86**

	**Total**	**36**	**25**	**337**	
